# Supplementary material for: Novel metrics to measure coverage in whole exome sequencing datasets reveal local and global non-uniformity
Source: Sci Rep. 2017 Apr 13;7:885. doi: 10.1038/s41598-017-01005-x (PMC5429826; doi:10.1038/s41598-017-01005-x)
Supplement: Supplementary file 1 — Supplementary Information [file 41598_2017_1005_MOESM1_ESM.doc]

# Novel metrics to measure coverage in whole exome sequencing datasets reveal local and global non-uniformity

# Qingyu Wang^1,2^, Cooduvalli S. Shashikant^1,3^, Matthew Jensen^1^, Naomi S. Altman^1,2^, Santhosh Girirajan^1,4,5*^

Table S1. WES and WGS datasets generated from different platform

| **Platforms** | **Capture kit** | **Sequencing technology** | **Sequencing read lengths** | **Dataset** | **# of samples** | **# of samples with average target coverage >75x (WES data)** | **Reference/database** |
| --- | --- | --- | --- | --- | --- | --- | --- |
| **NimbleGen** | SeqCap EZ Exome v2.0 kit | Illumina HiSeq | 50bp paired-end | 31 autism quad-families* | 110 | 108 | [^1^](#_ENREF_1) |
| **Agilent** | SureSelect All Exon v.2 Kit | Illumina HiSeq | 76bp paired-end | Patients with developmental brain disorders | 58 | 49 | dbGap [^2^](#_ENREF_2) (phs000492.v1.pa) |
| **Illumina** | TruSeq Exome Enrichment kit | Illumina Genome Analyzer IIx  Illumina HiSeq | 76bp paired-end  100 bp paired-end | Patients with leukemia  Embryonal tumor | 16 | 12 | SRA [^3^](#_ENREF_3)  SRP028277;  SRP032767 (WES samples only) |
| **WGS** | NA | Illumina HiSeq (TruSeq PCR-free preparation kit) | 250bp paired-end | PCR-free samples with average coverage > 30X | 26 | NA | 1000 Genome Project [^4^](#_ENREF_4) |

*The quad-families include one affected proband, one unaffected sibling and 2 parents.

Table S3. Number of genes in different coverage categories (classified by CCS score at 0.2) for different platforms by chromosome

| **Chromosomes** | **NimbleGen** | | | **Agilent** | | | **Illumina TruSeq** | | |
| --- | --- | --- | --- | --- | --- | --- | --- | --- | --- |
|  | CCS  ≤0.2 | CCS  >0.2 | Total | CCS  ≤0.2 | CCS  >0.2 | Total | CCS  ≤0.2 | CCS  >0.2 | Total |
| **1** | 1818 | 151 | 1969 | 1787 | 158 | 1945 | 1834 | 108 | 1942 |
| **2** | 1095 | 101 | 1196 | 1090 | 86 | 1176 | 1109 | 75 | 1184 |
| **3** | 976 | 58 | 1034 | 965 | 56 | 1021 | 1001 | 33 | 1034 |
| **4** | 662 | 45 | 707 | 662 | 50 | 712 | 664 | 38 | 702 |
| **5** | 784 | 56 | 840 | 764 | 55 | 819 | 784 | 46 | 830 |
| **6** | **809** | **168** | **977** | **812** | **168** | **980** | **858** | **139** | **997** |
| **7** | 777 | 86 | 863 | 740 | 100 | 840 | 796 | 57 | 853 |
| **8** | 584 | 57 | 641 | 564 | 77 | 641 | 582 | 47 | 629 |
| **9** | 672 | 74 | 746 | 653 | 88 | 741 | 682 | 54 | 736 |
| **10** | 628 | 77 | 705 | 622 | 69 | 691 | 648 | 53 | 701 |
| **11** | 1150 | 99 | 1249 | 1097 | 131 | 1228 | 1179 | 59 | 1238 |
| **12** | 928 | 61 | 989 | 910 | 64 | 974 | 939 | 47 | 986 |
| **13** | 280 | 28 | 308 | 276 | 27 | 303 | 273 | 28 | 301 |
| **14** | 530 | 49 | 579 | 508 | 66 | 574 | 548 | 33 | 581 |
| **15** | 505 | 48 | 553 | 503 | 50 | 553 | 512 | 34 | 546 |
| **16** | 660 | 125 | 785 | 642 | 134 | 776 | 709 | 65 | 774 |
| **17** | 993 | 135 | 1128 | 966 | 133 | 1099 | 1025 | 80 | 1105 |
| **18** | 234 | 25 | 259 | 235 | 20 | 255 | 240 | 16 | 256 |
| **19** | **1094** | **245** | **1339** | **992** | **327** | **1319** | **1183** | **148** | **1331** |
| **20** | 455 | 66 | 521 | 431 | 78 | 509 | 471 | 48 | 519 |
| **21** | 207 | 8 | 215 | 193 | 16 | 209 | 201 | 7 | 208 |
| **22** | 364 | 57 | 421 | 343 | 72 | 415 | 376 | 37 | 413 |
| **Total** | 16205 | 1819 | 18024 | 15755 | 2025 | 17780 | 16614 | 1252 | 17866 |

Table S4. Count of exons by percentage GC content and CCS score for different platforms

|  | GC < 20% | | 20%≤GC≤80% | | GC >80% | |
| --- | --- | --- | --- | --- | --- | --- |
|  | CCS >0.2 | Total | CCS >0.2 | Total | CCS >0.2 | Total |
| NimbleGen | 20 (13.6%) | 147 | 7286 (4.0%) | 183676 | 269 (92.1%) | 292 |
| Agilent | 18 (14.5%) | 124 | 13377 (7.4%) | 181719 | 258 (93.8%) | 275 |
| Illumina TruSeq | 18 (13.5%) | 133 | 6296 (3.5%) | 179269 | 151 (85.8%) | 176 |
| WGS | 0 (0%) | 170 | 122 (0.06%) | 189880 | 0 (0%) | 305 |

**Table S5. Library size, mapping and removed PCR duplicates of WES and WGS data sets**

| **Platforms** | **# of samples** | **Average library size** | **Average # of mapped reads** | **Average % mapped reads** | **Average % PCR duplicates** | **Average coverage** |
| --- | --- | --- | --- | --- | --- | --- |
| **NimbleGen** | 110 | 101,344,414 | 95,920,792 | 94.64% | 7.34% | 121.92X |
| **Agilent** | 58 | 41,294,491 | 41,035,677 | 99.37% | 0.31% | 93.75X |
| **Illumina** | 16 | 76,046,602 | 75,944,302 | 99.87% | 4.40% | 98.33X |
| **WGS** | 26 | 867,568,164 | 824,200,592 | 95.00% | N/A | 66.56X |

**Figure S1:** Histograms of the average coverage for all samples in each WES and WGS platform dataset. For WES platforms, the red line indicates the 75X coverage threshold we used to select samples for further analysis.


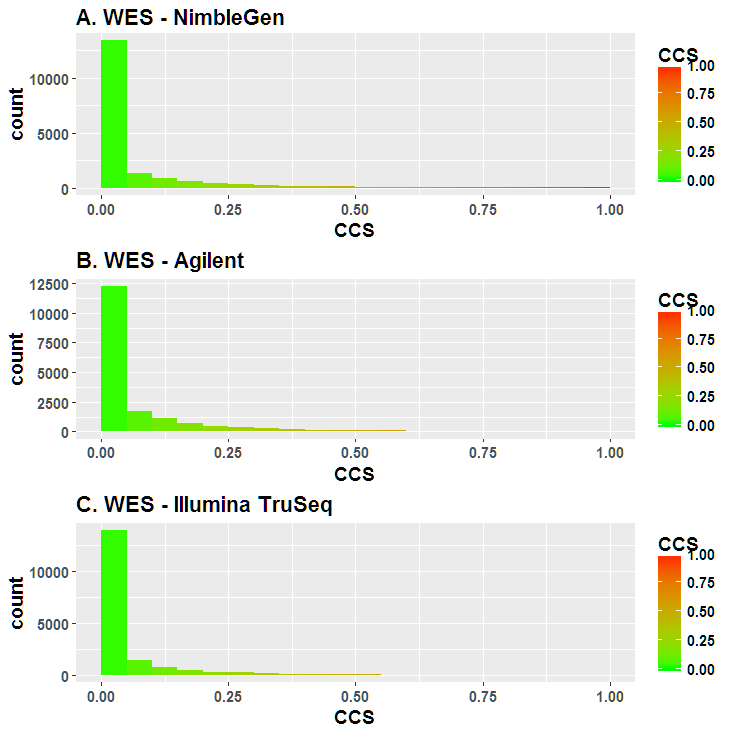


**Figure**S2: **The histograms show the distribution of CCS score of all genes in different WES platforms.** Note that there is skewness in the distribution towards the right suggesting variable CCS scores.

**Figure**S3: **Inconsistency in rank order of coverage of exons.** Plot of average coverage in neighboring exons of an example gene from different samples is shown. For example, the coverage of sample 1 is the best for exon 1, but this rank is not consistent throughout the gene for other exons (sample 1 is ranked 1^st^ on exon 1, ranked 3^rd^ on exon 2, ranked 3^rd^ on exon 3, ranked 2^nd^ on exon 4, ranked 2^nd^ on exon 5, and ranked 3^rd^ on exon 6).

**
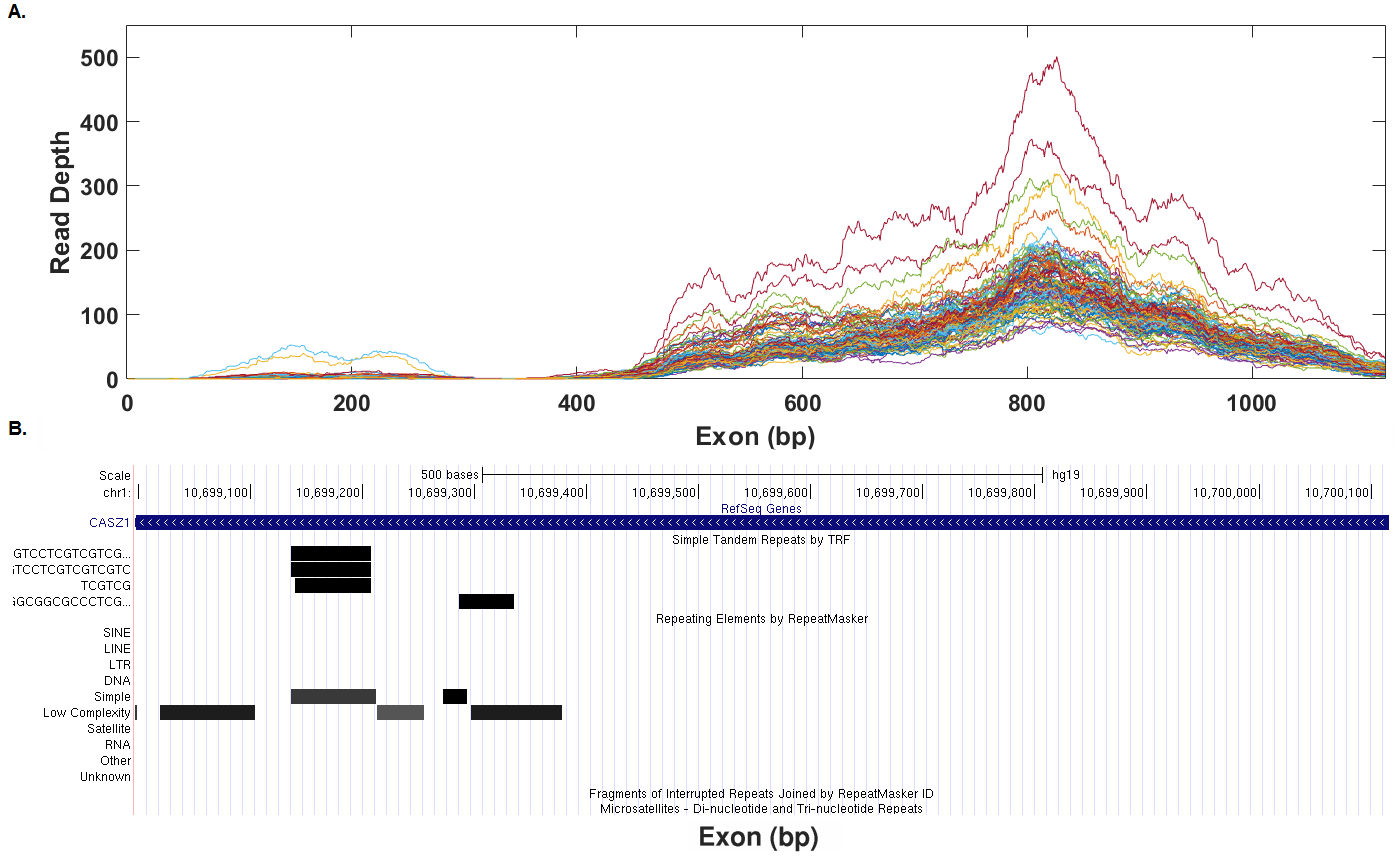
**

**Figure S4. Concurrence of repeat elements and coverage sparseness. (A)** The graph shows base coverage distribution along the length of the last coding exon of *CASZ1.* **(B)** UCSC browser screenshot is shown for the *CASZ1* genomic region.


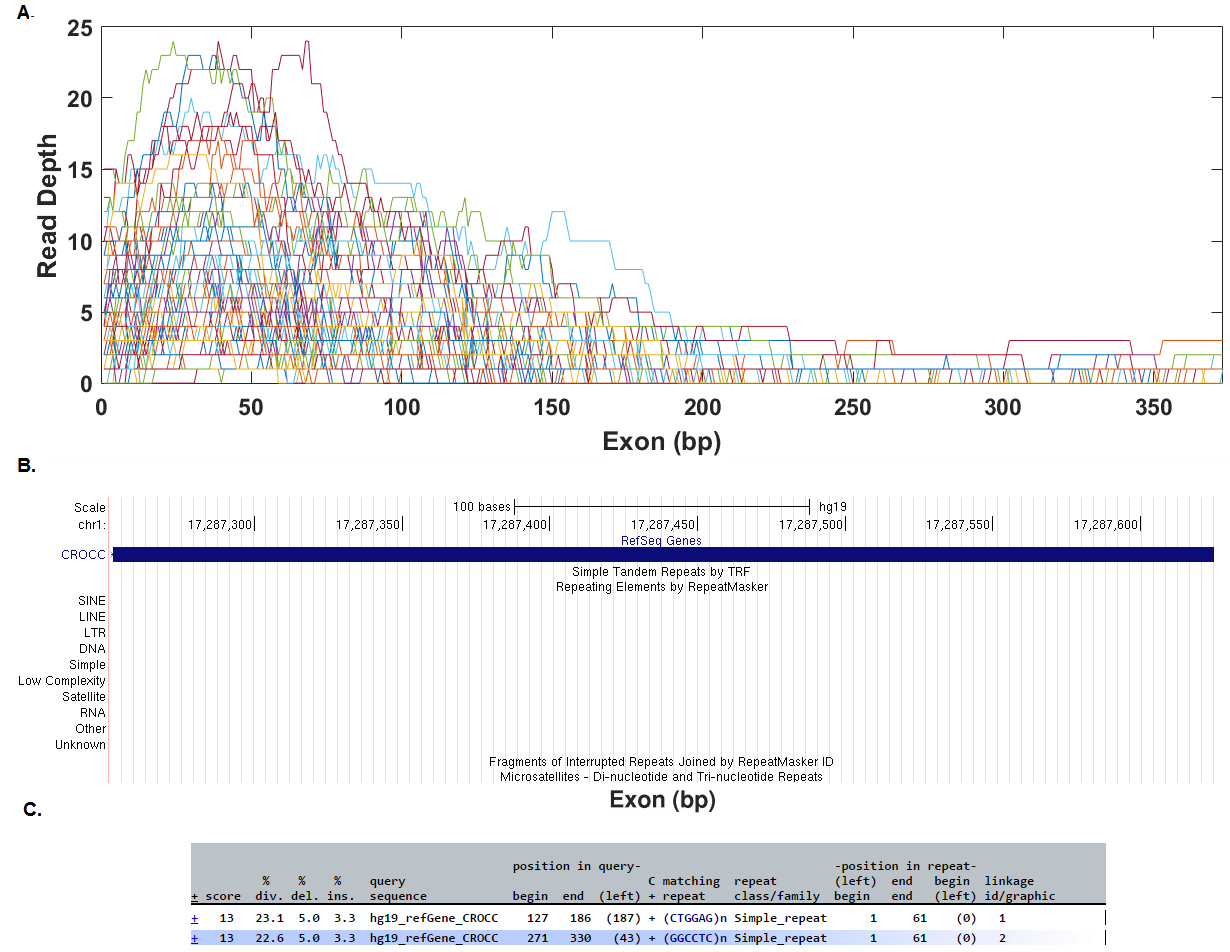


**Figure**S5: **Coverage sparseness regions of the exon in *CROCC* overlap with the repeat elements that are not annotated in the UCSC genome browser.** **A**. Base coverage distribution along the length of the 27^th^ coding exon of *CROCC* is shown. Different colors represent coverage of different samples. **B**. UCSC browser screen shot of the 27^th^ coding exon of *CROCC* genomic region shows absence of any repeats within the region. **C**. Identification of repeats when non-default parameters (“cross_match” for search engine and “slow” for speed/sensitivity) were used on RepeatMasker for the same region. The occurrence of repeat elements coincides with low coverage (note that the trough falls sharply as in **A**).

**Figure**S6: Density plots show GC content against cohort read depth of exons (calculated as CCS scores) for datasets generated from three different platforms.

**Figure**S7: The box plot shows cohort read depth coverage (CCS scores) of 57 autosomal genes reported in ACMG SF v2.0 (American College of Medical Genetics and Genomics). The following genes have at least one annotated pseudogene with high homology that may affect coverage caused by multiple mapping locations: *ATP7B, BMPR1A, FBN1, PKP2, PMS2, SHDB, SDHC, SDHD, SMAD3, TGFBR1,* and *TPM1*.

**Figure S8:** Scatter plots of the mappability (average alignability score) of each exon compared to CCS (top) and U_E_ (bottom) scores for the Agilent (left), NimbleGen (center) and TruSeq (right) WES platforms. R values were calculated from Pearson correlations.


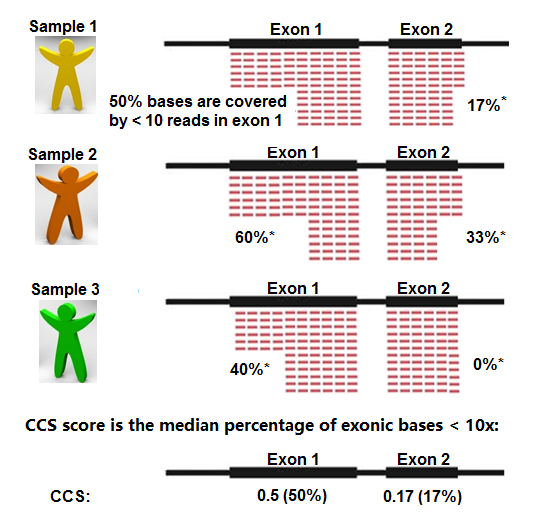


* Percentage of bases covered by < 10 reads in a specific exon

**Figure S9**: **Calculation of the Cohort Coverage Sparseness (CCS) scores for WES data generated from three samples**. The schematic shows red bars under each exon representing reads mapped to corresponding genomic locations. Note, in practice, mapped reads should overlap with each other.

**REFERENCES**

1 O'Roak, B. J. *et al.* Sporadic autism exomes reveal a highly interconnected protein network of de novo mutations. *Nature* **485**, 246-250, doi:10.1038/nature10989 (2012).

2 Mailman, M. D. *et al.* The NCBI dbGaP database of genotypes and phenotypes. *Nature genetics* **39**, 1181-1186, doi:10.1038/ng1007-1181 (2007).

3 Leinonen, R., Sugawara, H., Shumway, M. & International Nucleotide Sequence Database, C. The sequence read archive. *Nucleic acids research* **39**, D19-21, doi:10.1093/nar/gkq1019 (2011).

4 Genomes Project, C. *et al.* An integrated map of genetic variation from 1,092 human genomes. *Nature* **491**, 56-65, doi:10.1038/nature11632 (2012).

5 Kalia, S. S. *et al.* Recommendations for reporting of secondary findings in clinical exome and genome sequencing, 2016 update (ACMG SF v2.0): a policy statement of the American College of Medical Genetics and Genomics. *Genet Med* **19**, 249-255, doi:10.1038/gim.2016.190 (2017).

6 Karro, J. E. *et al.* Pseudogene.org: a comprehensive database and comparison platform for pseudogene annotation. *Nucleic acids research* **35**, D55-60, doi:10.1093/nar/gkl851 (2007).
